# Comparison of baseline lymphoma and HIV characteristics in Malawi before and after implementation of universal antiretroviral therapy

**DOI:** 10.1371/journal.pone.0273408

**Published:** 2022-09-01

**Authors:** Yolanda Gondwe, Evaristar Kudowa, Tamiwe Tomoka, Edwards D. Kasonkanji, Bongani Kaimila, Takondwa Zuze, Noel Mumba, Stephen Kimani, Maurice Mulenga, Fred Chimzimu, Coxcilly Kampani, Cara Randall, Amy Lilly, Satish Gopal, Yuri Fedoriw, Matthew Painschab

**Affiliations:** 1 University of North Carolina Project-Malawi, Lilongwe, Malawi; 2 University of Utah Huntsman Cancer Institute, Salt Lake City, UT, United States of America; 3 Kamuzu Central Hospital, Malawi Ministry of Health, Lilongwe, Malawi; 4 Department of Pathology and Laboratory Medicine, University of North Carolina, Chapel Hill, NC, United States of America; 5 National Cancer Institute Center for Global Health, Bethesda, MD, United States of America; 6 Lineberger Comprehensive Cancer Center, University of North Carolina, Chapel Hill, NC, United States of America; University of Kentucky, UNITED STATES

## Abstract

Access to antiretroviral therapy (ART) led to epidemiological changes in human immunodeficiency virus (HIV) associated lymphoma in high-income countries such as reductions in diffuse large B-cell lymphoma (DLBCL) and stable or increased Hodgkin lymphoma (HL) and Burkitt lymphoma (BL). In 2016, Malawi implemented a universal ART (UART) policy, expanding ART eligibility to all persons living with HIV (PLWH). We compare the distribution of lymphoma subtypes and baseline HIV and prognostic characteristics for lymphoma patients in Malawi before and after implementation of UART. We enrolled patients with pathologically confirmed incident lymphoproliferative disorders into a observational clinical cohort. At diagnosis, a comprehensive clinicopathological evaluation was performed. Of 412 participants, 156 (38%) were pre-UART (2013-June 2016) and 256 (62%) post-UART (July 2016–2020). HIV prevalence was 50% in both groups. The most common pre-UART diagnoses were DLBCL [75 (48%)], low-grade non-Hodgkin lymphoma (NHL) [19 (12%)], HL [17 (11%)] and, BL [13 (8%)]. For post-UART they were DLBCL [111 (43%)], NHL [28 (11%)], BL [27 11%)] and, HL [20 (8%)]. Among PLWH, 44 (57%) pre-UART initiated ART prior to lymphoma diagnosis compared to 99 (78%) post-UART (p = 0.02). HIV-ribonucleic acid was suppressed <1000 copies/mL in 56% (33/59) pre-UART and 71% (73/103) post-UART (p = 0.05). CD4 T-cell counts were similar for both groups. We observed similar findings in the subset of participants with DLBCL. Overall, there were no significant changes in incident lymphoma subtypes (p = 0.61) after implementation of UART, but HIV was better controlled. Emerging trends bear monitoring and may have implications for prognosis and health system priority setting.

**Trial registration: ClinicalTrials.gov identifier:** NCT02835911.

## Introduction

Human immunodeficiency virus (HIV) associated lymphoma has emerged as a leading cause of cancer-related mortality [1] for people living with HIV (PLWH) [2–4]. Africa has a high disease burden [5, 6] due to high HIV prevalence and patients often have advanced stage lymphoma and limited access to lymphoma treatment. In 2012, newly diagnosed cases of non-Hodgkin lymphoma (NHL) and Hodgkin lymphoma (HL) in Africa were estimated to be 36,800 and 7,900, respectively [5], with the prevalence of lymphoma cases projected to double by 2030 [3].

The advent of antiretroviral therapy (ART) led to changes in the epidemiology of HIV-associated lymphoma in high-income countries [2, 3, 7–12]. During the pre-ART-era, the risk of developing NHL in PLWH was 20–600 times higher than in the general HIV-negative population, with the risk of high-grade DLBCL being the highest (600-fold) [11, 13]. The risk of developing classical HL was also elevated 5–20 fold in PLWH [11]. Following widespread increase in ART coverage and use among PLWH, the risk of developing NHL decreased significantly, now estimated to be only 11–17 times greater than the general HIV-negative population [14]. In HICs, reductions in NHL were most prominent for aggressive NHL such as diffuse large B-cell lymphoma (DLBCL) and primary central nervous system lymphoma [7–11, 15]. Conversely, Burkitt lymphoma (BL) risk was not substantially affected and the risk of HL has remained stable or slightly increased following the expansion of ART [4, 11, 16].

The pathophysiology of HIV-associated lymphomas is heterogeneous. Although the etiological link between lymphoma and HIV is not fully understood, evidence shows that DLBCL occurs most commonly in individuals with advanced acquired immunodeficiency syndrome and severe immunosuppression [14], and is thus prevented by adherent ART use. In contrast, BL and HL occur more commonly in PLWH who have moderate immunosuppression [14]. Some evidence suggests that immune reconstitution inflammatory syndrome [17, 18] (a variety of inflammatory disorders that are uncovered in PLWH following improvements in CD4 T-cell counts) may be associated with HL development, potentially explaining the association between ART initiation and increased HL risk [17, 18].

In sub-Saharan Africa, available data is mostly inconsistent with trends observed in high-income countries. ART access has increased significantly in the past 15 years [19]. However, challenges including lack of high-quality cancer data, poor diagnostic infrastructure, and limited population-based cancer registries have resulted in a paucity of literature describing emerging epidemiological changes in HIV-associated lymphoma in the region [19]. A study in Uganda observed a 6% increase in high-grade NHL incidence for every 10% increase in ART coverage [20]. A study in Botswana observed a 10% increase in high-grade NHL incidence for every 10% increase in ART coverage [21]. In South Africa, similar trends were observed in a single-center cohort following the scale-up of ART, along with the emergence of formerly uncommon subtypes such as BL and plasmablastic lymphoma [22, 23]. In addition, no change in HL incidence was reported in Uganda [20], whereas an increase in HL incidence was reported in South Africa [24]. These studies, however, reported various limitations including retrospective design and incomplete ascertainment of HIV status and ART use, as well as improvement in access and quality of pathology services over time as a potential confounding factor.

In Malawi, HIV prevalence is 8.9% [25], making it a valuable setting to assess HIV-associated lymphoma. In 2016, Malawi became the third country in sub-Saharan Africa to implement a universal ART test and treat policy as per World Health Organization (WHO) 2015 HIV/AIDS guidelines [26]. The policy provides government-funded, lifelong ART for all PLWH in Malawi regardless of CD4 count, WHO staging, pregnancy status, or age [26, 27]. The estimated percentage of PLWH on ART in Malawi increased from 61% in 2016 to 88% in 2019 [25, 28], following the implementation of a national universal test and treat ART policy. We have previously published analyses [29–31] that compare clinical and pathological trends between HIV-associated lymphoproliferative disorders and non-HIV associated lymphoproliferative disorders that were enrolled into a single center prospective cohort of lymphoproliferative disorders at Kamuzu Central Hospital (KCH) in Lilongwe Malawi, one of two tertiary referral centers for cancer treatment in Malawi, serving approximately 10 million people. Herein, we compare the distribution of lymphoma subtypes for patients with newly diagnosed lymphoma, that were enrolled into the KCH Lymphoma Study, before and after the implementation of universal ART. We also compare baseline HIV characteristics (for PLWH) and prognostic characteristics of the lymphoma patients between the two periods. We seek to identify possible emerging changes in the clinicopathological presentation of lymphoma in our cohort amidst this policy shift, as well as compare our findings with existing findings from both high-income countries and other low-resource settings. We hypothesized that the proportion of HIV-associated DLBCL would be lower in the post-universal ART period compared to the pre-universal ART period. We also hypothesized that the proportion of HIV-associated HL and BL would increase slightly or remain the same between the two periods.

## Materials and methods

### Study design and population

The KCH Lymphoma Study is a prospective, observational cohort of lymphoma patients receiving treatment according to local standards of care in Lilongwe, Malawi. KCH is the main referral center for cancer treatment for the central and northern regions of Malawi, serving approximately 10 million people. The study was initiated in June 2013 and is one of the largest prospective cohorts of lymphoproliferative disorders in the region. The study was approved by the University of North Carolina Institutional Review Board and Malawi National Health Sciences Research Committee. All patients provide written informed consent prior to enrolment. Patients aged five and over with newly diagnosed pathologically confirmed lymphoproliferative disorders, who intend to complete treatment at KCH are eligible to be enrolled. All cases are diagnosed with real-time evaluation through weekly telepathology conferences involving two to five pathologists in Malawi and the United States. The pathologists render a consensus diagnosis using local tissue biopsy and immunohistochemistry as previously described [31, 32]. In addition, all specimens are shipped to University of North Carolina in the United States to confirm diagnoses with additional immunophenotyping using a larger panel of immunohistochemistry as needed.

### Procedures and analysis

At lymphoma diagnosis, a comprehensive baseline clinical and laboratory evaluation was performed for each participant, as described in detail previously [33]. Staging was completed by chest radiography, abdominal ultrasound, physical exam, and bone marrow biopsy. Tumor Epstein Barr virus status was assessed by Epstein-Barr encoding region (EBER) in situ hybridization and DLBCL cell-of-origin type was determined by the Hans immunohistochemical classifier [34]. Prior to June 2016, national HIV guidelines in Malawi restricted ART initiation to HIV-positive individuals with CD4 count ≤500 cells/μL, with WHO staging ≥III, who were pregnant or breastfeeding, or were younger than 5 years old. After June 2016, Malawi expanded eligibility for ART to anyone with a confirmed HIV infection [25, 26]. This report includes participants enrolled between June 2013 and July 2020. We categorized participants as pre-universal ART (pre-UART) if enrolled between June 2013-June 2016 or post-universal ART (post-UART) if enrolled between July 2016-July 2020. Among PLWH, we defined diagnosis of HIV prior to lymphoma as having been diagnosed with HIV at least three months before study enrollment. Likewise, among PLWH, we defined having been on ART prior to lymphoma as having been on ART for at least three months before study enrollment. Differences between pre-UART and post-UART participants were assessed using Pearson’s chi-squared test for categorical data, t-tests for normally distributed data and, Wilcoxon rank-sum test for non-normally distributed data. A two-sided alpha of 0.05 was considered statistically significant. Analyses were conducted using Stata version 12.0 (College Station, Texas, USA) [35], and figures were produced using the package ggplot2 [36] in R statistical package (Vienna, Austria, 2017) [37]. YG and EK conducted the primary data analysis and MSP created the figure. All authors had access to the primary clinical data.

### Role of the funding source

The funder of the study had no role in study design, data collection, data analysis, data interpretation, or writing of the report.

## Results

**Fig 1:**
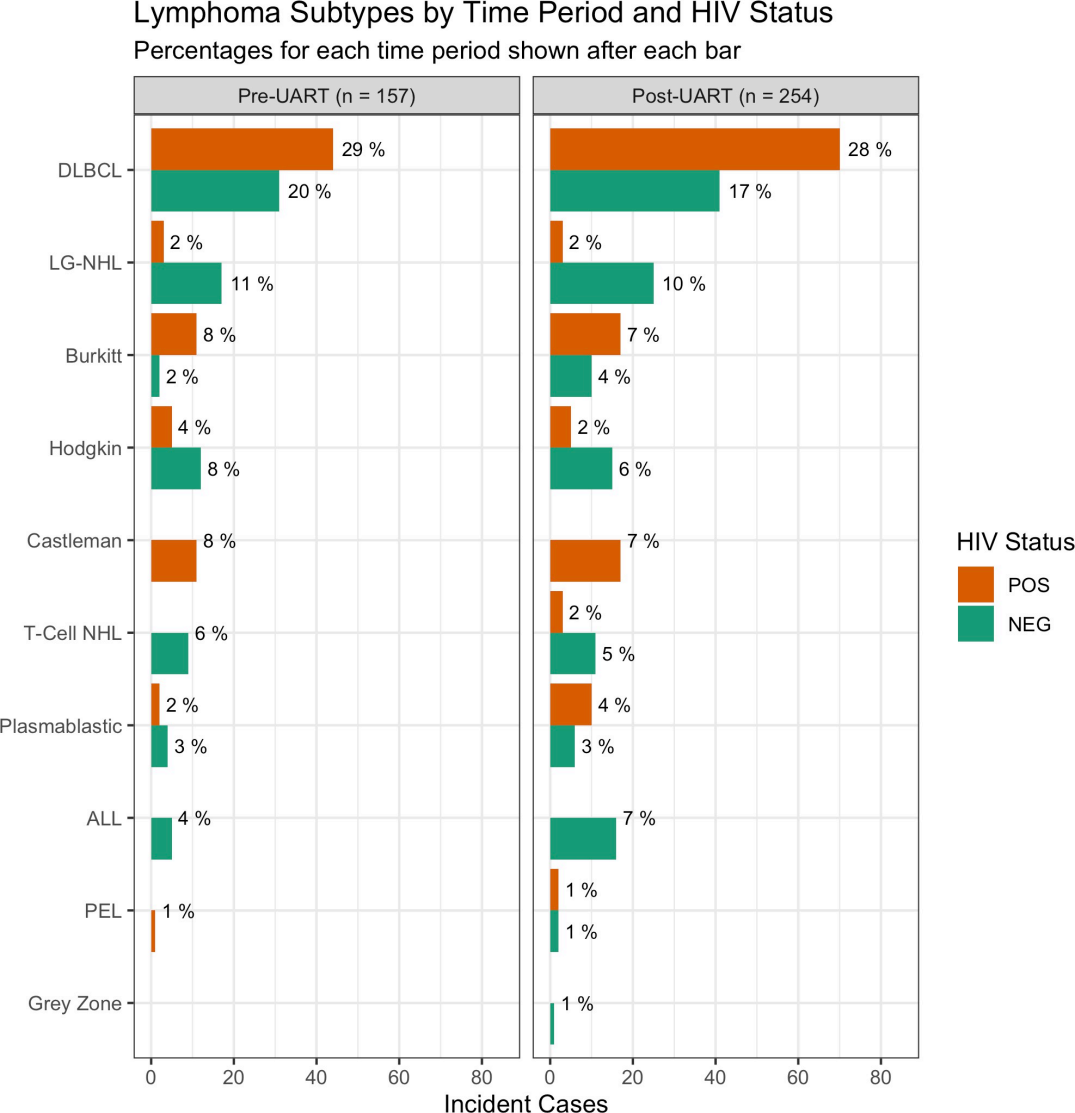
Distribution of lymphoma subtypes by HIV status in the pre-universal ART and post-universal ART period. Shown are absolute number of cases on the x-axis and percentage for the time period on the bar graph labels. *HIV status missing for one participant in post-UART. Abbreviations: pre-universal ART (pre-UART); post-universal ART (post-UART); Diffuse large B-cell lymphoma (DLBCL); low-grade non-Hodgkin’s lymphoma (LGNHL); T-cell non-Hodgkin’s lymphoma (T-Cell NHL); acute lymphoblastic leukemia/lymphoma (ALL); primary effusion lymphoma (PEL).

From 2013 to 2020, 412 participants were identified for the analytic cohort. One hundred and fifty-six (38%) participants were enrolled during the pre-UART period and 256 (62%) were enrolled during the post-UART period. The pathological distribution of lymphoma subtypes and HIV status is depicted in Fig 1. The most common diagnoses in the pre-UART group were DLBCL [75 (48%)], low-grade NHL [19 (12%)], HL [17 (11%)], BL [13 (8%)], and multicentric Castleman disease (MCD) [11 (7%)]. The most common diagnoses in the post-UART group were DLBCL [111 (43%)], low-grade NHL [28 (11%)], BL [27 11%)], HL [20 (8%)], and MCD [17 (7%)]. The differences in lymphoma subtypes between the pre and post-UART periods were not statistically significant (p = 0.61). Across the entire time period, PLWH were more likely to be diagnosed with DLBCL [114, (56%)], BL [28 (14%)] and MCD [28 (14%)] than HIV-negative participants [72 (35%) DLBCL, 12 (6%) BL, 0 (0%) MCD] (p<0.01 for all comparisons). A lower proportion of PLWH were diagnosed with HL [10 (5%)] and low grade NHL [6 (3%)] compared to HIV-negative participants [27 (13%) HL, 41 (20%) low grade NHL] (p<0.01 for both comparisons). The proportion of lymphoma subtypes among PLWH was similar between the pre-UART and post-UART period (p = 0.63). In the pre-UART PLWH subset, DLBCL accounted for 57% of lymphomas, while BL and HL accounted for 14% and 6% respectively. In the post-UART PLWH subset, DLBCL, BL and HL accounted for 55%, 13% and 4% of lymphomas respectively.

Baseline demographic and clinical characteristics of the cohort are depicted in Table 1, categorized by pre- and post-UART. The mean age of participants was 43 years (standard deviation (SD) 16), 256 (62%) participants were male, and 160 (42%) participants resided in Lilongwe, the urban capital of Malawi, with no significant differences between pre- and post-UART period. HIV prevalence was 50% in both groups (p = 0.93). Fewer PLWH in the pre-UART group [50 (67%)] were diagnosed with HIV prior to lymphoma diagnosis compared to PLWH in the post-UART group [103 (82%)] (p = 0.01). PLWH in the pre-UART had a median of only 21.2 months between HIV diagnosis and lymphoma diagnosis (interquartile range (IQR) 1.1, 69.5) compared to those in the post-UART who had a median of 34.4 months(IQR 4.6, 87.7)(p = 0.10).

**Table 1:** Baseline characteristics of the study population by pre- and post-universal ART groups.

	Pre-UART (n = 156)	Post-UART (n = 256)	P-value
**Age in years, mean(SD)**	44 (15)	43 (16)	0.51
**Male, n(%)**	101 (65)	155 (60)	0.39
**District, n(%)**			
Lilongwe	61 (39)	99 (39)	0.93
**HIV characteristics**			
**HIV Positive, n(%), (n = 204)**	77 (49)	127 (50)	0.93
**Diagnosed with HIV prior to enrollment, n(%)** (n = 200)	50 (67)	103 (82)	0.01
**Time since HIV diagnosis (months), median(IQR)**	21.2 (1.1, 69.5)	34.4 (4.6, 87.7)	0.10
**Initiated ART prior to enrollment, n(%),** (n = 200)	44 (57)	99 (78)	0.02
**Time on ART (months), median (IQR)**	16.8 (1.4, 60.8)	31.1 (5.8, 86.2)	0.02
**ART regimen, n(%),** (n = 143)			
TDF/3TC/EFV	37 (84)	59 (60)	<0.01
TDF/3TC/DTG	0 (0)	21 (21)	
Other	6 (13)	8 (8)	
Unknown	1 (2)	11 (11)	
**CD4 cells/μL, median(IQR)**	141.5 (82, 284)	156.0 (96, 273)	0.54
**Immunodeficiency Stage, n(%)**			
None or Not Significant (CD4 ≥ 500)	5 (8)	11 (10)	0.72
Mild-Advanced (CD4 200–499)	18 (27)	36 (31)	
Severe (CD4 <200)	43 (65)	68 (59)	
**HIV RNA (log copies/ml), median(IQR)**	2·4 (0, 4.2)	0 (0, 3.6)	0.07
**HIV RNA <1000 copies/mL, n(%)**	33 (56)	73 (71)	0.05

Shown are baseline demographic and clinical characteristics presented by pre and post-UART group. A p-value is shown in the last column to indicate the significance of the differences between groups.

**Abbreviations:** antiretroviral therapy (ART); human immunodeficiency virus (HIV); pre-universal ART (pre-UART); post-universal ART (post-UART); tenofovir/lamivudine/efavirenz (TDF/3TC/EFV); tenofovir /lamivudine/dolutegravir (TDF/3TC/DTG); cluster of differentiation 4 (CD4); ribonucleic acid (RNA); standard deviation (SD); interquartile range (IQR)

**Statistical significance:** Pearson’s chi-squared test for categorical data, t-tests for normally distributed data and Wilcoxon rank-sum test for non-normally distributed data.

PLWH in the pre-UART group had been on ART for a median of only 16.8 monthsat the time of enrollment (IQR 1.4, 60.8) compared to those in the post-UART group who were on ART for a median of 31.1 months(IQR 5.8, 86.2)(p = 0.02). In the pre-UART period, fewer PLWH [44 (57%)] were initiated on ART before lymphoma diagnosis compared to PLWH in the post-UART group [99 (78%)] (p = 0.02). Median CD4 T-cell count for PLWH at lymphoma diagnosis was 142 cells/μL (IQR 82–284) pre-UART and 156 cells/μL (IQR 96–273) post-UART (p = 0.54). PLWH in the post-UART period presented with lower HIV ribonucleic acid (RNA) [0 log copies/ml (IQR 0–3.6)] than those in the pre-UART period [2.4 log copies/ml (IQR 0–4.2)] (p = 0.07). Fewer PLWH in the pre-UART group [33 (56%)] had suppressed HIV RNA <1000 copies/ml compared to those in the post-UART period [73 (71%)] (p = 0.05).

Baseline clinical and pathological characteristics are depicted in Table 2 for a subset of 186 DLBCL participants, categorized by pre- and post-UART. One hundred and fourteen (61%) participants were HIV-positive. A slightly higher proportion of DLBCL participants were HIV-infected in the post-UART group (63%) compared to the pre-UART group (59%) (p = 0.54). Similar to the full cohort, in the pre-UART period, fewer PLWH [27 (64%)] were diagnosed with HIV prior to lymphoma diagnosis compared to the post-UART period [57 (83%)] (p = 0.03). Likewise, in the pre-UART period, fewer PLWH [22 (52%)] were initiated on ART prior to lymphoma diagnosis compared to the post-UART period [55 (80%)] (p = 0.02). Median CD4 T-cell count for PLWH at DLBCL diagnosis was 120 cells/μL (IQR 63–227) for pre-UART and 135 cells/μL (IQR 79–213) for post-UART (p = 0.52). Median HIV RNA for PLWH at DLBCL diagnosis was lower for post-UART patients [0 log copies/ml (IQR 0–4.2)] than for the pre-UART patients [3.0 log copies/ml (IQR 0–4.5)] (p = 0.04). In the pre-UART period, fewer PLWH [18 (50%)] had suppressed HIV RNA <1000 copies/ml compared to the post-UART period [39 (66%)] (p = 0.17). There were no significant differences in staging, Eastern Cooperative Oncology Group performance status, lactate dehydrogenase, duration of symptoms, presence of B-symptoms, or bone marrow involvement between the pre- and post-UART periods. Finally, Epstein-Barr virus positivity, cell of origin type, Ki67 proliferation index and age-adjusted Prognostic Index were similar in the pre- and post-UART periods.

**Table 2:** DLBCL subset baseline characteristics by pre- and post-universal ART groups.

	Pre-UART (n = 75)	Post-UART (n = 111)	p-value
**HIV characteristics**			
**HIV Positive, n(%)**	44 (59)	70 (63)	0.54
**Diagnosed with HIV prior to enrollment, n(%)** (n = 111)	27 (64)	57 (82)	0.03
**Time since HIV diagnosis (months), median(IQR)**	25 (1.1, 67.9)	37.4 (4.4, 100.5)	0.22
**Initiated ART prior to enrollment if HIV+, n(%)**	22 (52)	55 (80)	0.02
**Time on ART (months), median(IQR)**	20.6 (1.2, 56.6)	43.7 (10.9, 101.9)	0.02
**CD4 cells/μL, median(IQR)**	120 (63, 227)	135 (79, 213)	0.52
**Immunodeficiency stage, n(%)**			
None or Not Significant (CD4 ≥ 500)	0 (0)	4 (6.5)	0.31
Mild-Advanced (CD4 200–499)	11 (29)	16 (25)	
Severe (CD4 <200)	26(70)	42 (67)	
**HIV RNA (log copies/ml), median(IQR)**	3.0 (0, 4.5)	0 (0, 4.2)	0.04
**HIV RNA <1000 copies/ml, n(%)**	18 (50)	39 (66)	0.17
**Non-HIV clinical characteristics**			
**Ann Arbor Stage, n(%)**			
1–2	35 (47)	50 (51)	0.80
3–4	39 (53)	49 (49)	
**ECOG performance status, n(%)**			
≤1	42 (56)	63 (57)	0.83
≥2	33 (44)	47 (43)	
**Duration of symptoms (months), n(%)**			
≤3 months	25 (33)	42 (40)	0.67
>3 months	50 (67)	66 (61)	
**B-symptoms present, n(%)**	49 (66)	71 (72)	0.34
**Bone Marrow Involvement, n(%)**	5 (8)	5 (8)	0.81
**LDH ratio to upper limit of normal, median(IQR)**	1.9 (1.3, 3.2)	2.1 (1.4, 3.7)	0.22
**LDH above upper limit of normal, n(%)**	58 (83)	86 (87)	0.35
**EBV (EBER ISH) positive, n(%) n = 124**	8 (14)	7 (11)	0.54
**COO GC-type, n(%) n = 134**	39 (64)	41 (56)	0.36
**Ki67 index, median(IQR) n = 166**	80 (60, 90)	80 (60, 90)	0.86
**aaIPI, n(%)**			
0–1	22 (33)	37 (43)	0.27
2–3	46 (67)	49 (57)	

Shown are baseline clinical characteristics for a subset of DLBCL patients only, presented by pre-UART group and the post-UART group. A p-value is shown in the last column to indicate the significance level of the differences between pre-UART and post-UART results.

**Abbreviations:** antiretroviral therapy (ART); human immunodeficiency virus (HIV); pre-universal ART (pre-UART); post-universal ART (post-UART); cluster of differentiation 4 (CD4); ribonucleic acid (RNA); Eastern Cooperative Oncology Group performance status (ECOG performance status); lactate dehydrogenase (LDH); Epstein-Barr Virus (EBV); Epstein-Barr encoding region in situ hybridization (EBER ISH); cell of origin-germinal center type (COO GC-type); marker of proliferation (Ki67 index); Age-Adjusted International Prognostic Index (aaIPI); standard deviation (SD); interquartile range (IQR)

**Statistical significance:** Pearson’s chi-squared test for categorical data, t-tests for normally distributed data and Wilcoxon rank-sum test for non-normally distributed data.

## Discussion

A strong association between HIV and lymphoma has been noted since the beginning of the AIDS epidemic [9]. While high-income countries have observed significant changes in the epidemiology of HIV-associated lymphoma following the introduction and widespread use of ART [8, 9, 15, 16, 38, 39], few studies have evaluated the impact of increased ART access on lymphoma in sub-Saharan Africa. We have previously examined differences between lymphomas arising in HIV-infected and uninfected patients in our study [29–31]. Here, we examine the possible changes with respect to lymphoma subtypes and baseline characteristics for a single center cohort of newly diagnosed lymphoproliferative disorders in Malawi following the adoption of a universal test and treat ART policy. The percentage of PLWH on ART in Malawi increased from 61% in 2016 to 88% in 2019 [25, 28], following the implementation of a national universal test and treat ART policy. We hypothesized that the proportion of HIV-associated DLBCL would be lower in the post-UART period compared to the pre-UART period. We also hypothesized that the proportion of HIV-associated HL and BL would increase slightly or remain the same between the two periods.

The most common lymphoma subtypes identified in our study cohort were DLBCL, low-grade NHL and BL, for both the pre-UART and post-UART periods, consistent with results from a study by Mezger et al. [40] for a sub-Saharan Africa cohort derived from registries across ten countries. The proportion of DLBCL, HL and BL was similar between the pre and post-UART period, for both the full cohort and the subset of PLWH. These findings differ from trends reported in other sub-Saharan Africa studies. In particular, an increase in aggressive B-cell lymphoma was reported in South Africa, Uganda and Botswana following ART expansion [20–22]. This discrepancy may be attributed to confounding, concurrent changes in cancer diagnostic capabilities in those countries [21]. As ART coverage in sub-Saharan Africa has increased over time, cancer diagnostic capabilities in many settings have also simultaneously improved. As a result, more lymphoma cases are diagnosed, thus increasing lymphoma incidence despite ART scale-up. At our study site, we saw an increased number of cases overtime, however, there were no changes in lymphoma subtypes due to consistent high-quality diagnostic capability throughout the study period.

Our findings are also inconsistent with trends observed in the United States, where ART expansion was associated with declines in HIV-associated lymphoma [8, 41]. This can potentially be explained by the immunologic characteristics observed in our cohort. We observed encouraging improvements in HIV control among lymphoma patients during the post-UART period, including a significant increase in the proportion of lymphoma patients that were diagnosed with HIV and initiated ART prior to lymphoma diagnosis, as well as significant improvements in HIV RNA suppressions. These changes are likely at least partly attributable to the national ART policy change. There were, however, no improvements in CD4 T-cell count between the pre-UART and post-UART period. More than 50% of PLWH during both periods presented with severe immunodeficiency (CD4 <200) at baseline; this was low compared to other SSA cohorts [42] and other HICs [43]. Studies have shown that the rate of NHL remains elevated at lower CD4 T-cell counts [44], potentially accounting for to the lack of decline in DLBCL in our cohort following the implementation of universal ART. These results may highlight the need to accompany policies aimed at improving ART access with interventions aimed at improving early ART adherence, to prevent the development of deep and prolonged immunosuppression. Others have shown that CD4 T-cell count among PLWH on stable ART may remain low despite improvement in HIV RNA, indicating that CD4 T-cell count may be less reliable for monitoring long-term disease progress, response to ART, and long-term ART adherence compared to HIV RNA, especially in patients who are virologically suppressed [45, 46]. Persistently low CD4 counts are more common in patients who initiate ART at CD4 count <200 further supporting the need to identify all HIV-infected patients as early in the disease course as possible [47]. Epidemiological trends in HIV-associated malignancies may indeed be different in sub-Saharan Africa compared to high income countries if the proportion of patients presenting with low CD4 counts remains high.

Study strengths include high quality pathologically confirmed diagnoses using an innovative telepathology platform and recruitment of participants with prospective collection of data from the main cancer treatment facility in Malawi which serves approximately 60% of the country’s population. Our study also has some limitations. First and most critically, less than four years have elapsed from the start of the universal ART to the end of enrollment for this study. Therefore, it may be too early to reflect changes in lymphoma subtypes; more significant differences may be seen after more time has elapsed. Second, our study is based on a clinical cohort at a national teaching hospital. It is not reflective of a population-based cancer registry and therefore we cannot discern epidemiological trends that may exist at a national level. In addition, our cohort size is small compared to other studies that examine lymphoma epidemiology, limiting statistical power. Finally, our study lacks the ability to control for other cancer-related lifestyle risks such as tobacco use, diet, and alcohol consumption.

In conclusion, we found no significant differences in incident lymphoma subtypes in our study cohort when comparing the pre-UART and post-UART periods in Malawi. However, HIV control was improved among PLWH and lymphoma in the post-UART period but without significant improvement in baseline CD4 T-cell counts. Lymphoma remains a public health challenge in sub-Saharan Africa. As ART scale-up continues, it will be important to continue monitoring emerging epidemiologic trends related to lymphoma, as well as to continue efforts to provide early and effective ART to PLWH to reduce lymphoma risk and decrease cancer-attributable morbidity and mortality.
